# Counselling versus low-intensity cognitive behavioural therapy for persistent sub-threshold and mild depression (CLICD): a pilot/feasibility randomised controlled trial

**DOI:** 10.1186/s12888-015-0582-y

**Published:** 2015-08-15

**Authors:** Elizabeth Freire, Christopher Williams, Claudia-Martina Messow, Mick Cooper, Robert Elliott, Alex McConnachie, Andrew Walker, Deborah Heard, Jill Morrison

**Affiliations:** Federal University of Juiz de Fora, Research Center in Spirituality and Health - NUPES School of Medicine, Av. Eugênio do Nascimento s/n°, Bairro Dom Bosco, CEP: 36038-330 Juiz de Fora, MG Brazil; Psychosocial Psychiatry, Institute of Health and Wellbeing, University of Glasgow, Administration Building Gartnavel Royal Hospital, 1055 Great Western Road, Glasgow, G12 0XH UK; Robertson Centre for Biostatistics, University of Glasgow, Boyd Orr Building University Avenue, Glasgow, G12 8QQ UK; Department of Psychology, University of Roehampton, Holybourne Avenue, London, SW15 4JD UK; Counselling Unit, School of Psychological Sciences & Health, University of Strathclyde, GH507 Graham Hills Building, 40 George Street, Glasgow, G1 1QE UK; General Practice and Primary Care, Institute of Health and Wellbeing, University of Glasgow, 1 Horselethill Road, Glasgow, G12 9LX UK

## Abstract

**Background:**

Persistent depressive symptoms below the threshold criteria for major depression represent a chronic condition with high risk of progression to a diagnosis of major depression. The evidence base for psychological treatments such as Person-Centred Counselling and Low-Intensity Cognitive Behavioural Therapy for sub-threshold depressive symptoms and mild depression is limited, particularly for longer-term outcomes.

**Methods:**

This study aimed to test the feasibility of delivering a randomised controlled trial into the clinical and cost effectiveness of Low-Intensity Cognitive Behavioural Therapy versus Person-Centred Counselling for patients with persistent sub-threshold depressive symptoms and mild depression. The primary outcome measures for this pilot/feasibility trial were recruitment, adherence and retention rates at six months from baseline. An important secondary outcome measure was recovery from, or prevention of, depression at six months assessed via a structured clinical interview by an independent assessor blind to the participant’s treatment condition. Thirty-six patients were recruited in five general practices and were randomised to either eight weekly sessions of person-centred counselling each lasting up to an hour, or up to eight weeks of cognitive-behavioural self-help resources with guided telephone support sessions lasting 20–30 minutes each.

**Results:**

Recruitment rate in relation to the number of patients approached at the general practices was 1.8 %. Patients attended an average of 5.5 sessions in both interventions. Retention rate for the 6-month follow-up assessments was 72.2 %. Of participants assessed at six months, 71.4 % of participants with a diagnosis of mild depression at baseline had recovered, while 66.7 % with a diagnosis of persistent subthreshold depression at baseline had not developed major depression. There were no significant differences between treatment groups for both recovery and prevention of depression at six months or on any of the outcome measures.

**Conclusions:**

It is feasible to recruit participants and successfully deliver both interventions in a primary care setting to patients with subthreshold and mild depression; however recruiting requires significant input at the general practices. The evidence from this study suggests that short-term Person-Centred Counselling and Low-Intensity Cognitive Behaviour Therapy are potentially effective and their effectiveness should be evaluated in a larger randomised controlled study which includes a health economic evaluation.

**Trial registration:**

Current Controlled Trials ISRCTN60972025.

## Background

Depression is a major public health problem that is associated with poor quality of life, impaired interpersonal and family relationships, occupational disadvantage, residual disability, and suicide [1, 2]. Having persistent depressive symptoms below the threshold criteria for major depression is a chronic and disabling condition with a high risk of progression [3, 4]. Depressive symptoms are considered ‘persistent’ if they have been present for several months or continue despite active monitoring by a clinician or low-intensity intervention [5].

Low-Intensity Cognitive Behavioural Therapy (Li-CBT), involving guided self-help CBT interventions, has been found to be an effective intervention for mild to moderate depression compared with no treatment controls, with a mean effect (Cohen’s *d*) of 0.8 [6]. Guided self-help CBT, which is termed a low intensity (LI) intervention because the amount of practitioner time is limited compared to traditional high intensity (HI) expert-led treatments, can be delivered through books, classes, computers and online resources [7]. Expert delivered CBT and guided self-help CBT showed equivalent outcomes for depression in a recent meta-analysis of findings from randomised controlled trials [8].

Person-centred counselling (PCC), also known as Rogerian psychotherapy or non-directive counselling, has been the most common psychological intervention offered in community settings in the United Kingdom (UK) [9]. PCC aims to provide an empathic, genuine, and accepting therapeutic relationship that fosters clients’ inner capacities and resources, promoting positive change [10]. A review of the effectiveness of counselling in primary care found that counselling was superior to standard General Practitioner (GP) care with an effect size of 0.5 to 0.6, with the strongest effects in the short term [11].

However, in spite of the two approaches being recommended for mild to moderate depression in the UK by the National Institute for Health and Care Excellence (NICE) [5] there is no evidence of comparative effectiveness of PCC and Li-CBT in patients with sub-threshold and mild depression. Therefore, this study aimed to test the feasibility of delivering a randomised controlled trial (RCT) into the clinical and cost effectiveness of Low-Intensity Cognitive Behaviour Therapy (Li-CBT) versus Person-Centred Counselling (PCC) for patients with persistent sub-threshold depressive symptoms and mild depression.

## Methods

This study was a two-arm, parallel group, pilot randomised trial comparing short-term (six months) outcomes of PCC and Li-CBT. It was approved by the West of Scotland Research Ethics Service (WoSRES) at National Health Service Greater Glasgow and Clyde (NHS GG&C), REF: 12/WS/0173. This study is registered with Current Controlled Trials (ISRCTN60972025) and the protocol has been published [12].

### Recruitment

Participants were identified and screened for eligibility at five general practices in Glasgow (UK) according to recruitment methods developed for the study [12]. Inclusion criteria were: Age ≥ 16; scoring 5–18 on the Patient Health Questionnaire (PHQ-9) [13] (i.e. mild or moderate low mood) at screening; screened positive for persistent (i.e. > 6 months) sub-threshold depressive symptoms or mild depression (SCID) [14]; and capable of taking part in research procedures, as assessed by the researcher at baseline assessment. Exclusion criteria were: Alcohol/substance dependence; receiving other psychological intervention; bipolar disorder; bereavement as the presenting issue; Post-traumatic Stress Disorder (PTSD); cognitive impairment; unable to understand, speak, read or write in English; terminal illness; and unable to take part in any of the interventions.

When patients arrived at the general practice, they received a pack with study information and a self-report measure for depression (PHQ-9) which they were asked to complete. The pack also included a questionnaire about reasons for not wanting to take part in the study. Participants scoring 5–18 on the PHQ-9 (i.e. mild or moderate depression) were considered potentially eligible for the study. The list of potentially eligible participants identified at the practice was subsequently checked by the GP to identify anyone who met the exclusion criteria for the study. Those potentially eligible participants, not screened out by the GP, were invited to attend a baseline assessment with a researcher. Eligibility to enter the trial was then assessed by the researcher using a structured clinical interview (SCID). We aimed to recruit 50 patients into the pilot study.

### Randomisation

At the end of the baseline assessment, all eligible and consenting participants were randomly allocated by the researcher to one of the two treatments through a remote telephone randomisation system, after entering the necessary baseline participant information. Randomisation was produced by a computer-generated code. The use of an automated telephone randomisation system ensured concealment of allocation. Participants were randomised individually in blocks of four, stratified by practice to balance practice level effects.

### Interventions

Patients randomised to PCC were offered eight weekly, 50 min, sessions of person-centred counselling delivered by qualified counsellors. At minimum, they had a diploma-level training in PCC. A therapy manual was designed specifically for this trial based on the Skills for Health competence framework for Humanistic Psychological Therapies [15].

Patients randomised to Li-CBT received a range of written CBT self-help booklets and worksheets [16] supported by an optional linked online support course. Participants received telephone support over a series of up to eight support sessions lasting 20–40 min. Guidance was delivered by trained support staff working with a community charity organisation.

### Outcome measures

Participants attended face-to-face assessments with a researcher at baseline, 3 and 6 month follow-ups.

The primary outcome measures of the study were recruitment, adherence and retention rates at six months from baseline. Secondary outcome measures were: (1) changes at 6 months on the GRID Hamilton Depression Rating Scale (GRID-HAMD-17) [17]; (2) recovery from, or prevention of, depression according to DSM-IV (Diagnostic and Statistical Manual of Mental Disorders, fourth edition) [18] diagnosis at 6 months assessed via the Structured Clinical Interview for DSM-IV (SCID); (3) changes at 6 months on the Patient Health Questionnaire–9 (PHQ-9), Work and Social Adjustment Scale (WSAS) [19], Euroquol (EQ-5D-5 L) [20], and SF12v2 MH Enhanced [21]. Participants were also asked to complete the Client Satisfaction Questionnaire (CSQ-8) [22] at 3-month follow-up assessment. The 6-month follow-up assessments were carried out by an independent assessor blind to the participant’s treatment.

The feasibility of collecting data for an economic evaluation was tested by asking participants to complete, at all assessment points (i.e., baseline, 3 and 6 month follow-ups), (i) an adapted version of the Client Service Receipt Inventory (CSRI) [23]; (ii) questions about medicines use; (iii) EQ-5D-5 L, a general quality of life measure suitable for calculating QALYs (Quality-adjusted Life-years).

### Adherence/competence checks

All sessions were audio-recorded and a random selection was checked for adherence/competence. Two independent raters analysed recordings of telephone support sessions of Li-CBT using the Guided CBT Rating Scale [14]. This uses a 3-point scale, ranging from ‘0’ to ‘2’ , where ‘1’ corresponds to the minimal acceptable level of competence/adherence. Two independent raters analysed segments of recordings of counselling sessions using the Person-Centred and Experiential Psychotherapy Scale (PCEPS) [24]. This uses a 10-item, 6-point scale, ranging from ‘1’ to ‘6’ , where ‘3.5’ corresponds to the minimal acceptable level of competence/adherence.

### Statistical analysis

Continuous variables were summarised as mean and standard deviation (SD). They were compared between groups using t-tests, and within groups using paired t-tests. Results of t-tests were reported as mean difference with 95 % confidence intervals (CIs) and *p*-values. For effect sizes, Hedges’ g [25] were given along with 95 % CIs. All continuous variables were approximately normally distributed.

Categorical variables were summarised as number and percentage per group. They were compared between groups using exact Fisher tests. In reporting effect sizes, Cramér’s V [26] were given. Recruitment and retention rates were reported with 95 % (CIs) calculated by the Clopper and Pearson method [27] with effect sizes, reported using Cohen’s h [28] . All analyses were carried out in R version 3.0.1 [29].

## Results

### Primary outcome: recruitment feasibility

**Fig. 1:**
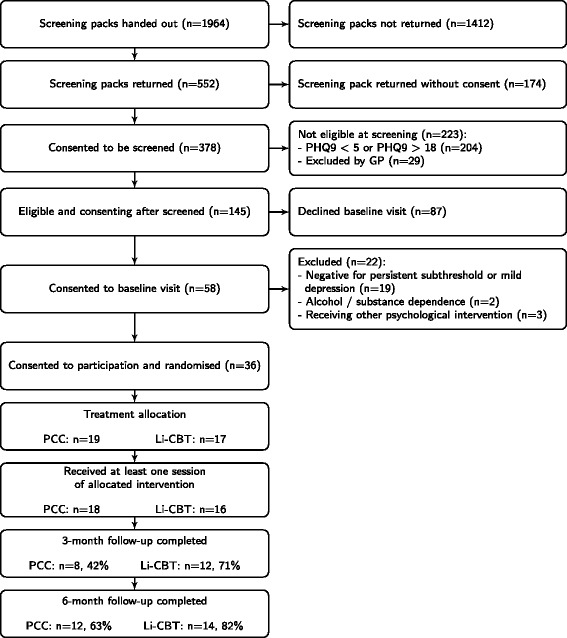
CONSORT Flow Diagram

Thirty-six eligible and consenting participants were randomised to one of the two treatments as depicted in Fig.  1 between February and November 2013. We had originally aimed to recruit 50 participants to the study. Demographics of the randomised population are presented at Table 1*.*

**Fig. 2:**
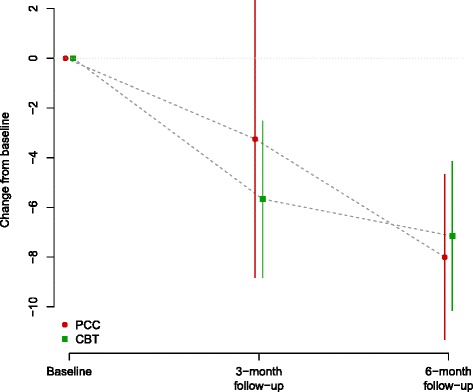
GRID-HAMD-17 - Mean and confidence interval for change from baseline at 3-month and at 6-month follow up, by treatment group

**Table 1:** Demographics in randomised population

		All	PCC	Li-CBT
N		36	19	17
Age at baseline		44.0 (17.8)	43.3 (15.7)	44.6 (20.4)
Gender	Male	6 (16.7 %)	2 (10.5 %)	4 (23.5 %)
	Female	30 (83.3 %)	17 (89.5 %)	13 (76.5 %)
Marital status	Single/Unmarried	15 (41.7 %)	8 (42.1 %)	7 (41.2 %)
	Married/Partnership	14 (38.9 %)	8 (42.1 %)	6 (35.3 %)
	Separated/Divorced	3 (8.3 %)	2 (10.5 %)	1 (5.9 %)
	Widow/Widower	4 (11.1 %)	1 (5.3 %)	3 (17.6 %)
Education	Secondary	6 (17.6 %)	2 (11.8 %)	4 (23.5 %)
	Higher/Further	25 (73.5 %)	13 (76.5 %)	12 (70.6 %)
	Other	3 (8.8 %)	2 (11.8 %)	1 (5.9 %)
Ethnic group	White	35 (97.2 %)	18 (94.7 %)	17 (100.0 %)
	Asian	1 (2.8 %)	1 (5.3 %)	0 (0.0 %)
Living situation	Alone	9 (25.0 %)	4 (21.1 %)	5 (29.4 %)
	Partner	8 (22.2 %)	5 (26.3 %)	3 (17.6 %)
	Children	6 (16.7 %)	5 (26.3 %)	1 (5.9 %)
	Partner and Children	5 (13.9 %)	2 (10.5 %)	3 (17.6 %)
	Other	8 (22.2 %)	3 (15.8 %)	5 (29.4 %)
Employment status	Paid or self employment	20 (62.5 %)	12 (66.7 %)	8 (57.1 %)
	Unemployed	4 (12.5 %)	2 (11.1 %)	2 (14.3 %)
	Housewife/husband	1 (3.1 %)	1 (5.6 %)	0 (0.0 %)
	Retired	5 (15.6 %)	3 (16.7 %)	2 (14.3 %)
	Exempt through disability	2 (6.2 %)	0 (0.0 %)	2 (14.3 %)

Data summarised as mean (standard deviation) or number (percent) per category

The overall recruitment rate in relation to screening packs distributed in the general practice clinics was 36/1,964 = 1.83 %. The recruitment rate in relation to the number of screening packs returned (*N* = 552) was calculated as 6.5 % [4.6, 8.9]. The overall rate of screening packs returned in relation to the screening packs handed out was 28.1 %. We received 216 completed questionnaires with reasons for declining participation in the study (Table 2).

**Table 2:** Reasons for declining participation - numbers and percentage of all who declined (*N* = 216)

Reason	N (%)
None ticked	5 (2.3 %)
No low mood or depression	171 (79.2 %)
Too busy	58 (26.9 %)
Does not want to take part in research study	62 (28.7 %)
Does not want to have counselling	69 (31.9 %)
Does not want to have guided CBT	57 (26.4 %)
Other	13 (6.0 %)

Two participants (one in each arm) withdrew their participation in the study before they received any treatment. In the PCC arm, participants attended an average of 5.4 (SD 3.0) counselling sessions and participants in the Li-CBT arm took part in an average of 5.5 (SD 1.7) telephone support sessions. Twenty participants completed the 3-month follow-up assessments (retention rate 55.6 % [38.1, 72.1]) and 26 participants completed the 6-month follow-up assessments (retention rate 72.2 % [54.8, 85.5]). See Table 3 and Fig. 2.

**Table 3:** Retention rates (in %) at 3 and 6 months, with 95 % confidence intervals and *p*-values comparing randomised groups from Fisher’s Exact Test

	All	Randomised Group		*p*-value	Cohen's h
		PCC	Li-CBT		
3-month follow-up retention rate	55.6 (38.1, 72.1)	42.1 (20.3, 66.5)	70.6 (44.0, 89.7)	*p* = 0.106	−0.583
6-month follow-up retention rate	72.2 (54.8, 85.8)	68.4 (43.4, 87.4)	76.5 (50.1, 93.2)	*p* = 0.717	−0.181

**Fig. 3:**
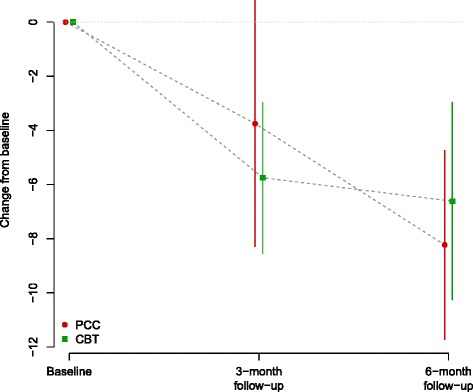
PHQ - Mean and confidence interval for change from baseline at 3-month and at 6-month follow up, by treatment group

Compared to baseline, the mean scores on GRID-HAMD-17 changed significantly at 6 months in both arms (See Table 4 and Fig. 3). The mean change for the PCC arm was −8.0 [−11.3, −4.7], p < 0.001, *g =* 1.57 [0.70, 2.44] and the mean change for the Li-CBT arm was −7.2 [−0.2, −4.2], p < 0.001,, *g =* 1.23 [0.38, 2.09]. The between treatment group comparison was not significant (mean difference −0.8, *p* = 0.7).

**Table 4:** GRID-HAMD-17. Data summaries at baseline, 3 and 6 months

		All	Randomised Group		Between-group difference (Li-CBT – PCC)
			PCC	Li-CBT	
Baseline					
	Mean (SD)	14.7 (4.8)	16.1 (4.1)	13.2 (5.1)	
3 months					
	Mean (SD)	9.5 (6.5)	12.2 (8.0)	7.7 (4.8)	
Change from baseline	Estimate (95 % CI)	−4.7 (−7.4, −2.0)	−3.2 (−8.8, 2.3)	−5.7 (−8.8, −2.5)	2.4 (−3.6, 8.4)
Hedges' g	Estimate (95 % CI)	0.94 (0.35, 1.54)	0.67 (−0.26, 1.59)	1.09 (0.23, 1.95)	0.41 (−0.61, 1.43)
	*p*-value	*p* = 0.001	*p* = 0.211	*p* = 0.002	*p* = 0.398
6 months					
	Mean (SD)	7.7 (5.1)	8.8 (5.0)	6.7 (5.2)	
Change from baseline	Estimate (95 % CI)	−7.6 (−9.7, −5.5)	−8.0 (−11.3, −4.7)	−7.2 (−10.2, −4.2)	−0.8 (−5.1, 3.4)
Hedges' g	Estimate (95 % CI)	1.40 (0.82, 1.98)	1.57 (0.70, 2.44)	1.23 (0.38, 2.09)	−0.16 (−1.00, 0.69)
	*p*-value	*p* < 0.001	*p* < 0.001	*p* < 0.001	*p* = 0.684

Estimated changes from baseline at each follow-up visit, on original scale and as effect sizes (Hedge’s g) with 95 % confidence intervals and *p*-values from paired t-tests. Estimated between-group differences at each follow-up point with 95 % confidence intervals and *p*-values from two-sample t-tests of changes from baseline

At 6 months, ten (71.4 %) participants who had a diagnosis of mild depression at baseline (*n* = 14) had recovered; and eight (66.7 %) participants assessed at 6-month follow-up who had a diagnosis of persistent subthreshold depression at baseline (*n* = 12) had not developed major depression (see Table 5). There was no significant difference between treatment groups for both recovery (*p* = 0.7) and prevention of depression at 6 months (*p* = 1.0).

**Table 5:** Diagnosis at 6-month follow up, overall and in relation to diagnosis at baseline, with p-values comparing randomised groups from Fisher’s Exact Test

		All	Randomised Group		*p*-value	Cramér's V
			PCC	Li-CBT		
Diagnosis in those with major depressive episode at baseline	Neither Persistent subthreshold depression Major depressive episode	10 (71.4 %)	6 (66.7 %)	4 (80.0 %)	*p* = 0.760 ^F^	0.28
		2 (14.3 %)	2 (22.2 %)	0 (0.0 %)		
		2 (14.3 %)	1 (11.1 %)	1 (20.0 %)		
Diagnosis in those with persistent subthreshold depression at baseline	Neither Persistent subthreshold depression Major depressive episode	8 (66.7 %)	2 (50.0 %)	6 (75.0 %)	*p* = 1.000 ^F^	0.20
		2 (16.7 %)	1 (25.0 %)	1 (12.5 %)		
		2 (16.7 %)	1 (25.0 %)	1 (12.5 %)		
Diagnosis in all	Neither Persistent subthreshold depression Major depressive episode	18 (69.2 %)	8 (61.5 %)	10 (76.9 %)	*p* = 0.832 ^F^	0.18
		4 (15.4 %)	3 (23.1 %)	1 (7.7 %)		
		4 (15.4 %)	2 (15.4 %)	2 (15.4 %)		

^F^Fisher-test

**Fig. 4:**
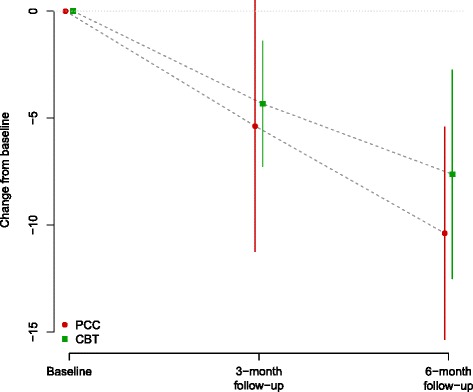
WSAS - Mean and confidence interval for change from baseline at 3-month and at 6-month follow up, by treatment group

Compared to baseline, the mean scores on PHQ-9 (depression) and WSAS (social function) changed significantly indicating a decrease in depressive symptoms and an improvement in social and work-related functioning at 6 months (*p* < 0.001) in both arms, and again there was no significant difference between treatment group for both measures (see Tables 6 and 7 and Fig. 4). The Hedges’ g pre-post effect size for PHQ-9 for the two groups combined was 1.25 [0.68, 1.82]; the comparable Hedges’ g for the WSAS was 1.12 [0.55, 1.69]; both effect sizes are considered 'large' according to current convention.

**Table 6:** PHQ-9. Data summaries at baseline, 3 and 6 months

		All	Randomised Group		Between-group difference (Li-CBT – PCC)
			PCC	Li-CBT	
Baseline					
	Mean (SD)	12.8 (5.2)	15.2 (4.0)	10.2 (5.2)	
3 months					
	Mean (SD)	7.5 (5.7)	10.9 (7.1)	5.2 (3.3)	
Change from baseline	Estimate (95 % CI)	−5.0 (−7.2, −2.7)	−3.8 (−8.3, 0.8)	−5.8 (−8.5, −3.0)	2.0 (−3.0, 7.0)
Hedges' g	Estimate (95 % CI)	0.97 (0.37, 1.57)	0.82 (−0.11, 1.76)	1.06 (0.20, 1.91)	0.40 (−0.62, 1.42)
	*p*-value	*p* < 0.001	*p* = 0.092	*p* = 0.001	*p* = 0.401
6 months					
	Mean (SD)	6.0 (5.7)	7.5 (6.0)	4.4 (5.1)	
Change from baseline	Estimate (95 % CI)	−7.4 (−9.8, −5.1)	−8.2 (−11.7, −4.7)	−6.6 (−10.3, −3.0)	−1.6 (−6.4, 3.2)
Hedges' g	Estimate (95 % CI)	1.25 (0.68, 1.82)	1.51 (0.65, 2.38)	1.09 (0.25, 1.93)	−0.26 (−1.11, 0.58)
	*p*-value	*p* < 0.001	*p* < 0.001	*p* = 0.002	*p* = 0.493

Estimated changes from baseline at each follow-up visit, on original scale and as effect sizes (Hedge’s g) with 95 % confidence intervals and p-values from paired t-tests. Estimated between-group differences at each follow-up point with 95 % confidence intervals and p-values from two-sample t-tests of changes from baseline

**Table 7:** WSAS. Data summaries at baseline, 3 and 6 months

		All	Randomised Group		Between-group difference (Li-CBT – PCC)
			PCC	Li-CBT	
Baseline					
	Mean (SD)	16.9 (6.8)	18.3 (7.1)	15.2 (6.2)	
3 months					
	Mean (SD)	11.9 (7.2)	13.6 (5.9)	10.8 (8.1)	
Change from baseline	Estimate (95 % CI)	−4.8 (−7.3, −2.2)	−5.4 (−11.3, 0.5)	−4.3 (−7.3, −1.4)	−1.0 (−7.2, 5.2)
Hedges' g	Estimate (95 % CI)	0.70 (0.11, 1.28)	0.67 (−0.25, 1.60)	0.61 (−0.21, 1.43)	−0.18 (−1.19, 0.84)
	*p*-value	*p* = 0.001	*p* = 0.067	*p* = 0.008	*p* = 0.719
6 months					
	Mean (SD)	8.7 (7.7)	8.8 (4.9)	8.7 (10.2)	
Change from baseline	Estimate (95 % CI)	−9.1 (−12.3, −5.8)	−10.4 (−15.4, −5.4)	−7.6 (−12.5, −2.7)	−2.8 (−9.3, 3.8)
Hedges' g	Estimate (95 % CI)	1.12 (0.55, 1.69)	1.47 (0.61, 2.33)	0.79 (−0.05, 1.62)	−0.33 (−1.20, 0.54)
	*p*-value	*p* < 0.001	*p* = 0.001	*p* = 0.006	*p* = 0.395

Estimated changes from baseline at each follow-up visit, on original scale and as effect sizes (Hedge’s g) with 95 % confidence intervals and p-values from paired t-tests. Estimated between-group differences at each follow-up point with 95 % confidence intervals and p-values from two-sample t-tests of changes from baseline

Participants’ overall satisfaction with the treatment received as measured by the CSQ-8 was high (Mean score 25.8, S 6.7, *N* = 22), (range of the instrument: 8–32). The mean CSQ-8 score for participants in the Li-CBT arm was 27.8 (SD 6.4, *N* = 13) and for participants in the PCC arm was 22.9 (SD 6.4, *N* = 9), *p* = 0.093.

### Economic analysis

EQ-5D-5 L showed a pattern of change consistent with the depression-specific instruments (PHQ-9 and GRID-HAMD-17), with scores above normal at baseline but close to normal at 3 and 6 months (See Table 8). The data collected with the modified CSRI form showed the expected phenomenon of outlying values. In the CSRI question ‘Have you used any of these services in the last 3 months?’ , none of the respondents reported any contacts with social workers, psychologists, psychiatrists, community support workers, and mental health nurses. In relation to the question ‘Please list below any drugs taken over the past one month’ , of all medicines listed, only 6.6 % were medicines prescribed for depression and/or low mood.

**Table 8:** EQ-5D-5 L. Data summaries at baseline, 3 and 6 months

		All	Randomised Group		Between-group difference (Li-CBT – PCC)
			PCC	Li-CBT	
Baseline					
	Mean (SD)	0.67 (0.21)	0.66 (0.19)	0.69 (0.23)	
3 months					
	Mean (SD)	0.76 (0.13)	0.77 (0.10)	0.75 (0.15)	
Change from baseline	Estimate (95 % CI)	0.09 (0.04, 0.15)	0.13 (0.01, 0.24)	0.07 (0.00, 0.14)	0.05 (−0.07, 0.18)
Hedges' g	Estimate (95 % CI)	−0.43 (−1.01, 0.14)	−0.58 (−1.50, 0.34)	−0.30 (−1.11, 0.51)	0.42 (0.60, 1.44)
	p-value	*p* = 0.003	*p* = 0.032	*p* = 0.049	*p* = 0.369
6 months					
	Mean (SD)	0.75 (0.17)	0.79 (0.10)	0.70 (0.22)	
Change from baseline	Estimate (95 % CI)	0.12 (0.05, 0.18)	0.16 (0.05, 0.28)	0.07 (−0.01, 0.15)	0.10 (−0.04, 0.23)
Hedges' g	Estimate (95 % CI)	−0.37 (−0.89, 0.16)	−0.78 (−1.56, 0.01)	−0.07 (−0.85, 0.71)	0.57 (−0.29, 1.43)
	*p*-value	*p* = 0.001	*p* = 0.007	*p* = 0.095	*p* = 0.146

Estimated changes from baseline at each follow-up visit, on original scale and as effect sizes (Hedge’s g) with 95 % confidence intervals and *p*-values from paired t-tests. Estimated between-group differences at each follow-up point with 95 % confidence intervals and p-values from two-sample t-tests of changes from baseline

### Adherence/competence checks

For the Li-CBT arm, 60 % of the support workers had a sample of their sessions checked for adherence/competence. The mean score for all rated sessions in the Guided CBT Rating Scale was 1.50 (*N* = 8; SD 0.14; range: 1.20–1.67), corresponding to an acceptable level of competence/adherence. In the PCC arm there were five counsellors. Four (80 %) of the counsellors had a sample of their sessions checked for adherence/competence. One counsellor fell below the minimal level of competence/adherence, obtaining a mean score of 2.89 on the PCEPS. This counsellor delivered the intervention to three participants in the study. The mean score for the rated sessions of the other three counsellors was 4.23 (SD 0.41; range: 3.79 - 4.86), which corresponds to an acceptable level of competence/adherence.

## Discussion

The evidence base for counselling and low-intensity CBT interventions for sub-threshold depressive symptoms and mild depression is limited, particularly in relation to longer-term outcomes. This feasibility/pilot study is the first step towards the development of a full-scale comparative trial that could report short and long-term outcomes (including cost-effectiveness) of these interventions.

Recruitment of patients into studies of depression is a well-documented challenge [30]; therefore the information about recruitment, adherence and retention rates obtained in this study is useful in designing a full-scale randomised controlled trial. This study demonstrated that the method of recruitment used was costly in terms of time and effort, and it did not achieve its recruitment target of 50 patients. Only 36 patients were recruited to the study via 1964 screening packs distributed. The adherence rates for patients who began treatment were good and the follow-up/retention rate at six months was similar to the follow-up rate at this time in other studies [31, 32]. However, for a definitive RCT, we would recommend utilising wider recruitment methods, such as notices in local media sites and routes for self-referral.

On the other hand, our findings suggest that both PCC and Li-CBT are associated with large reductions in persistent subthreshold and mild depression. Pre-post effect sizes for the GRID-HAMD-17 at 3- and 6-month follow-up ranged from 0.67 to 1.57. However, a comparative study would need to be powered to detect much smaller between-group differences, whether designed to show equivalence/non-inferiority or superiority. A realistic differential effect size for a definitive trial would be 0.3, which would require a sample size of 235 per group. In this study, we achieved 70 % follow-up at 6 months, implying that approximately 670 participants would have to be randomised.

In relation to the economic evaluation, the data collected with the modified CSRI could be reduced for a full-scale RCT, without losing any meaningful detail. EQ-5D-5 L was sufficiently responsive to change indicating that it could be used in a definitive RCT.

The involvement of the voluntary sector in delivering the telephone support for the Li-CBT was innovative and provided valuable experience of working with this sector that can be utilised in future studies and health service interventions. Adherence/competence checks were successfully carried out in a sample of recordings of sessions for each treatment by independent raters. Problems with adherence/competence in the PCC condition underscore the importance of checking adherence. In a larger, definitive trial, we would check adherence early on during the delivery of the interventions to assess the need for further training.

A two-arm design was adopted rather than a three-armed study with a control (treatment as usual) condition after discussion with the funder who recommended that a two–arm study would have lower cost and more power to compare the relative cost-effectiveness of the interventions. In retrospect, the lack of a control group was a weakness of this study since it was possible that improvement in either or both arms reflected the impact of time, usual care or other factors. Therefore, we recommend that for a full-scale RCT, a usual care arm be offered to address the question of what level of improvement would occur over time with no additional active intervention being offered. An added advantage is that this would allow usual care to be described in detail.

## Conclusions

This pilot study has provided important information for the design of a full-scale randomised controlled trial. A key question from the current study is how to detect and then support people whose symptoms are at such a low level that their motivation to engage and complete treatment may be low. We found that it is possible to detect potential participants; however this required significant input at the GP surgery. We also found that it was possible to successfully deliver interventions using PCC or Li-CBT. The evidence from this study suggests that short-term Person-Centred Counselling and Low Intensity Cognitive Behavioural Therapy are potentially effective with this client population and that their effectiveness should be subject to a larger randomised controlled study.

## Abbreviations

CBT: Cognitive behavioural therapy
CI: Confidence interval
CSRI: Client service receipt inventory
DSM: Diagnostic statistic manual of mental disorders
GP: General practitioner
GRID-HAMD-17: GRID Hamilton rating scale for depression
IQR: Interquartile range
Li-CBT: Low-intensity cognitive behavioural therapy
NHS: National health service
QALY: Quality-adjusted life-year
PCC: Person-centred counselling
PCEPS: Person-centered and experiential psychotherapy scale
PHQ-9: Patient health questionnaire
PTSD: Post-traumatic stress disorder
RCT: Randomised controlled trial
SCID: Structured clinical interview for DSM disorders
SD: Standard deviation
UK: United Kingdom
WSAS: Work and social adjustment scale
